# An interview with Greg J. Huang

**DOI:** 10.1590/2177-6709.20.6.032-036.int

**Published:** 2015

**Figure:**
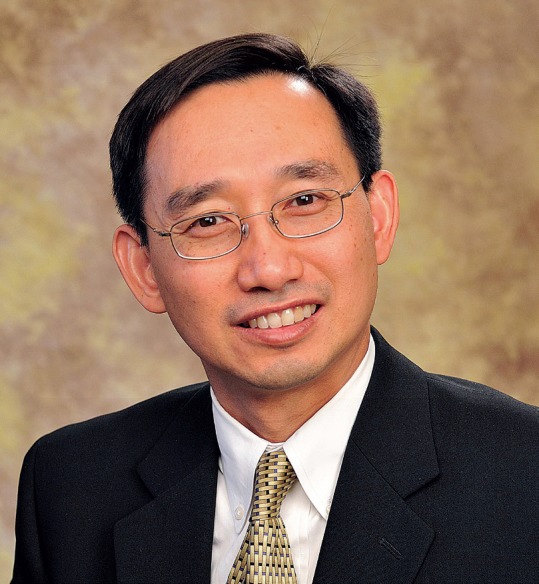

While I was a PhD reciprocal exchange student at University of California, Los Angeles (UCLA), in 2011, I had the pleasure to meet great names in international Orthodontics. I was introduced to Dr. Greg Huang, a brilliant professor and an extraordinary human being.. In addition to having a brilliant clinical and academic career, Dr. Huang is also chair of one of the most renowned Orthodontics programs worldwide: at the University of Washington.

Undoubtedly, thanks to Dr. Huang, Orthodontics has followed scientific evidence-based guidelines, therefore leaving a number of merely clinical assumptions behind. Once our honored guest has been duly introduced, I express my sincere gratitude to my colleagues Steven Lindauer, Luciane Menezes, Weber Ursi and Marcos Janson for accepting my invitation to participate actively in this interview. I am also thankful for having been given the honor by Dental Press to conduct such an experience. I wish readers have a reading experience as pleasing and valuable as the scientific path that brought us here.

Durante o meu doutorado sanduíche na Universidade da Califórnia em Los Angeles (UCLA), em 2011, tive a satisfação de entrar em contato com grandes nomes da Ortodontia internacional. Entre esses, foi-me apresentado, pelo Dr. Carlos Jorge Vogel, um brilhante professor e uma pessoa extraordinária, o Dr. Greg Huang. Por trás de uma brilhante carreira clínica e acadêmica, o Dr. Huang é, atualmente, chefe de um dos programas de Ortodontia mais respeitados do mundo, na Universidade de Washington. Com certeza, graças ao Dr. Huang, a Ortodontia vem buscando diretrizes baseadas em evidências científicas e, assim, deixando para trás muitos "achismos" clínicos. Feitas as devidas apresentações do nosso ilustre entrevistado, dedico meus cordeais agradecimentos aos colegas Steven Lindauer, Luciane Menezes, Weber Ursi e Marcos Janson, por aceitarem o convite para participar ativamente dessa entrevista. Agradeço, ainda, a Dental Press, pela honra, a mim concedida, de conduzir essa experiência. Desejo a todos os leitores que essa leitura seja tão prazerosa e rica quanto o trajeto científico que nos trouxe a essa entrevista.

André Wilson Machado

## Could you please provide us with some of your dental/orthodontic background? Andre Wilson Machado

I went to Dental School at the University of Florida ('87), and then to the University of Washington for my Master of Science in Dentistry and Certificate in Orthodontics (both in '89). I was in private practice in Florida for 10 years. During that time, I taught one day each month at the University of Florida. In 1999, I returned to the University of Washington as an Acting Assistant Professor. I also enrolled in the Master of Public Health program, and completed my MPH in Epidemiology, in 2001. I became an Associate Professor in 2005, and then a Full Professor in 2011. In 2008, I was selected as Chair of our Department.

What is the approach that you and your faculty take in educating new orthodontists at the University of Washington? In other words, what is the university teaching philosophy? Steven Lindauer, Andre W. Machado

I believe our education could be described as evidence-based, open-minded, outcomes-focused, and patient-centered. We have tried to steer clear of orthodontic "dogma", and we place a high emphasis on reading the literature critically. Clinically, we have many affiliate faculties, all of whom are encouraged to teach graduate students their own particular philosophy and techniques. We feel that exposure to many facets of Orthodontics best prepares our students for real world practice.

## How have your focus and interests changed regarding Orthodontics and orthodontic academics during the time you have spent as a member of the faculty? Steven Lindauer

When I initially decided to focus on an academic career, my intention was primarily to teach clinically. I never thought that my training in Epidemiology would lead to a career that has become increasingly involved with clinical research. In the past, we primarily conducted retrospective, single-center studies at the University of Washington. Now, of course, prospective studies carried out at multiple sites or in research networks are gaining traction. I have been extremely fortunate to conduct clinical studies in a regional practice-based research network that operated from 2005-2012, and now, in a national practice-based network.

I also never imagined that I would have the opportunity to collaborate with so many talented colleagues from all over the world. In my various roles with the University of Washington, the American Association of Orthodontists (AAO), orthodontic journals, and several textbooks, I have had the opportunity to be exposed to many interesting facets of our profession. 

## What is an evidence-based practice in Orthodontics and how you became interested in it? Can you also talk about your remarkable book entitled "Evidence-based Orthodontics" and how this book may help clinicians? Steven Lindauer, Andre W. Machado

As defined by the American Dental Association (ADA), an evidence-based approach is based on three important elements: a clinician's education and experience; the scientific literature; and a patient's values, preferences, and unique condition. All three of these must be considered in arriving at treatment that is most appropriate for an individual.

I became interested in evidence-based practice due to my training in Epidemiology. Many principles of evidence-based Medicine were developed by two physician/epidemiologists (Archie Cochrane, in the UK, and David Sackett, in Canada). As you know, our literature is full of studies that report opposing findings, leaving us confused and wondering what to believe. Evidence-based Dentistry provides a framework from which to objectively evaluate our literature, and in my opinion, knowing how to employ evidence-based methods is an invaluable skill that all practitioners should master. Fortunately, the guidelines to perform systematic reviews and meta-analyses have continued to evolve and improve, and reviewers are also doing a better job identifying well-conducted reviews for publication. Thus, the systematic reviews and meta-analyses that are being published currently are usually of high quality. 

Our textbook, Evidence-based Orthodontics, was a very enjoyable project for Steve Richmond, Kate Vig, and me. We were fortunate to have many talented orthodontists from around the world contribute by assembling information on evidence-based methods, as well as by summarizing the evidence on many important orthodontic topics. We are looking forward to working on a second edition in the near future.

## Recently, evidence-based practice has been gaining ground in Orthodontics. Following this trend, what is your opinion about self-ligating brackets? What are the pros/cons? Marcos Janson, Weber Ursi

The recent evidence on self-ligating brackets seems to indicate that they reduce chair time, but probably do not decrease alignment time or treatment time significantly.^1^^-^ 8 Given this information, there is nothing wrong with using self-ligating brackets. However, I do believe we should be careful not to imply that they are associated with shorter treatment time or superior results, based on the current evidence. 

## What is your point of view on the use of functional appliances to stimulate mandibular growth? And how about the use of fixed mandibular propulsion devices? Marcos Janson, Luciane Menezes

Because so many patients have Class II malocclusions due to mandibular retrognathia, it makes sense that orthodontists would like to enhance mandibular growth with functional appliances. While this appears to happen to some degree when assessing immediately after treatment, very little additional growth seems to be maintained long term.^9^ A recent systematic review on fixed functional appliances^10^ indicates that their mechanism of action is similar to removable functional appliances,^11^ i.e., dentoalveolar, rather than skeletal. However, fixed functional appliances have the advantage of being compliance independent; thus, they may be more efficient than removable functional appliances.

## What is the evidence of the association between different malocclusions, orthodontic treatment, and temporomandibular disorders (TMD)? Luciane Menezes, Marcos Janson

I do not consider myself an expert in the field of TMD, but except for some unique individuals or in some extreme malocclusions, TMD seems to be minimally affected by malocclusion.^12^ One entire issue of the AJODO was dedicated to reporting on studies investigating the relationship between Orthodontics and TMD in 1992, and the studies largely reported no associations.^13^ Several systematic reviews have investigated the relationship between occlusal adjustment and TMD, finding little evidence that occlusal adjustment can prevent or cure TMD.^14^^,^ 15 ^,^ 16 One randomized trial performed at the University of Washington found that inexpensive mouthguards were as effective as custom splints for addressing TMD. And neither of these two therapies was superior to self-care with no splint.^17^ There are many publications with conflicting evidence on prevention and treatment of TMD. I would suggest that randomized trials, systematic reviews, and meta-analyses provide our best, least biased, knowledge on this topic.

## The association of third molars and Orthodontics has been extensively studied over the years. What are the indications for third molar extractions in evidence-based clinical practice? Weber Ursi

We conducted a practice-based study on third molar removal in the USA.^18^^,^ 19 To a very large extent, third molars were recommended for extraction for prophylactic reasons. Only in about 12% of our sample of 16-22 year-olds was there some pathologic condition. I believe there are many instances in which third molars should be removed - impaction, crowding, recurrent pericoronitis, etc. However, I do believe that we should evaluate all third molars before referring them for removal. 

## What are the most important factors to consider in terms of post-treatment stability in adult patients? Marcos Janson

Relapse in adult patients can occur quickly, and retention should be part of the treatment planning process. In general, tooth movement is more difficult in adults, and, therefore, more robust methods must be considered for the retention phase. For example, I am more likely to use bonded retainers in adults with significant irregularity. Similarly, bonded retainers may be indicated to assist with holding extraction spaces closed. From our post-retention sample at the University of Washington, we have found that irregularity increases with each decade of life, so our practice of long-term retention seems well justified. Perhaps in the future we will have some biological agents or new techniques to stabilize teeth in their corrected positions. 

## Based on your vast experience with open bite cases, can you briefly discuss the most important aspects to increase the stability of treated patients with this malocclusion? Luciane Menezes, Andre W. Machado

The literature on open bite treatment is voluminous, but the literature on open bite stability is significantly less. What we seem to know is that surgical treatment may have a little higher rate of long-term stability than orthodontic treatment.^20^ However, this is a challenging comparison, as most surgical treatment is performed in adults, and many of the studies investigating the stability of orthodontic treatment for open bite malocclusions include only adolescent patients. If we wish to improve stability, it makes sense to try to eliminate habits as early as possible. Extractions may also assist with closure and stability.^21^ Currently, we are conducting a large, multi-site study to investigate treatment and stability in adult anterior open-bite patients. We should have some additional information to report on stability in a few years. 

## What is a clinically effective protocol to avoid white spot lesions in orthodontic treatment with fixed appliances? Weber Ursi

Based on the most recent Cochrane review, fluoride varnish applied every 6 weeks may reduce the occurrence of white spot lesions 70%.^22^ Other types of fluoride application have also been shown to be effective, such as fluoride rinse in a recent randomized controlled trial.^23^ Of course, conscientious oral hygiene should not be discounted, but it seems like white spot lesions continue to occur in at least some of our patients. Other potential factors, such as salivary flow and diet, are perhaps under investigated as risk factors. 

## What would you say has been the most rewarding aspect of your job and why would you say that? Steven Lindauer

In my career as an orthodontic educator, perhaps the most rewarding aspect is playing a role in the education and development of our young orthodontists. It is always exciting to welcome a new class of students, and to see their progression in skills and knowledge during the time they are at the University of Washington. 

## Based on your brilliant carrier in Orthodontics, what is the most important piece of advice you would give to residents in Orthodontics around the world? Andre W. Machado

I would suggest to them three things. First, take full advantage of the learning opportunities that are afforded to you during your training. Unless you pursue an academic career, most of you will never be in such a rich, intense, and diverse educational environment. Learn from your faculty and your peers, try as many techniques as possible, and keep an open mind to new ideas. Assess your results honestly. 

Second, educate yourself in the science of our profession. Learn about evidence-based methods, keep up with the literature, and attend meetings. Employ an evidence-based approach to your daily practice by using the best evidence to guide your clinical recommendations. 

Third, always place your patient's best interests first. Recommend the same treatments to them that you would recommend to your own family members.
